# Differential diagnosis for a mandibular mass – a rare case of an odontoameloblastoma in a red deer (*Cervus elaphus elaphus*)

**DOI:** 10.1186/s12917-021-02759-3

**Published:** 2021-01-28

**Authors:** Svenja Hartung, Kernt Köhler, Christiane Herden, Manfred Henrich

**Affiliations:** Institute of Veterinary Pathology, Faculty of Veterinary Medicine, Justus-Liebig-University, Frankfurter Straße 96, 35392 Gießen, Gießen, Germany

**Keywords:** Red deer (*Cervus elaphus elaphus*), Odontoameloblastoma, Odontogenic tumor, Oral cavity

## Abstract

**Background:**

Mandibular masses caused by inflammatory processes due to bacterial infections, most common with *Actinomyces bovis*, are well known in herbivors. This case represents a rare differential diagnosis to common inflammatory processes which cannot be distinguished from neoplasia without detailed histopathological examination.

**Case presentation:**

A large unilateral mandibular mass of a free-ranging female adult red deer (*Cervus elaphus elaphus*) was submitted for pathological examination. The animal had been shot due to its poor body condition. Grossly, the mandibular mass showed gingival ulceration and necrosis. Histologically, irregular strands and islands of odontogenic epithelial cells and a matrix of dentin and osteoid-like material were found, leading to the diagnosis of an odontogenic tumor. Considering the animal’s age the tumor was classified as odontoameloblastoma with secondary chronic purulent osteomyelitis.

**Conclusions:**

Odontogenic tumors are rare in domestic and wildlife species and so far have not been reported in red deer. In addition to the more common inflammatory processes of the mandibula and other neoplastic diseases of the oral cavity, odontogenic tumors represent a rare differential diagnosis that must be kept in mind especially when masked by inflammatory lesions.

## Background

 Mandibular masses can occasionally occur in domestic and wildlife species such as the Bennett-Wallaby. Differential diagnoses for mandibular masses in herbivors include inflammatory and neoplastic processes affecting the mandibular bone and the surrounding tissue including oral structures like the gingiva and the periodontium. Odontogenic tumors are classified depending on the presence or absence of mesenchymal components beside the involvement of odontogenic epithelium [1]. Benign neoplasms of odontogenic origin consisting of all types of odontogenic tissue such as dentin, enamel, and odontogenic epithelium are termed odontoma. Compound odontomas show odontogenic epithelium in combination with enamel and dentin formation in organized denticles. Complex odontomas contain irregular arrangements of odontogenic ectomesenchyme (i.e. mesenchyme of dental pulp (positive for vimentin), cemental matrix, dentinal matrix, enamel matrix) combined with odontogenic epithelium [1]. Ameloblastic fibro-odontomas are characterized by smaller numbers of epithelial cells embedded in a well differentiated loose collagenous mesenchyme with hard dental tissue formation [1]. In veterinary medicine, most odontomas are found in young animals, so that they are rather thought to be a developmental anomaly or a hamartoma [1, 2]. Odontoameloblastoma are characterized by the prescence of odontogenic epithelilial cells, small amounts of odontogenic mesenchyme and the production of dentin and in some cases enamel in close relationship to the odontogenic epithelium [3]. In ameloblastic fibro-odontoma a higher amount of odontogenic ectomesenchyme and a loose collagenous stroma would be expected, although it is an important differential diagnosis to odontoameloblastoma. In human medicine ameloblastic fibro-odontoma and complex odontoma are discussed to be one entity [4].

## Case presentation

**Fig. 1:**
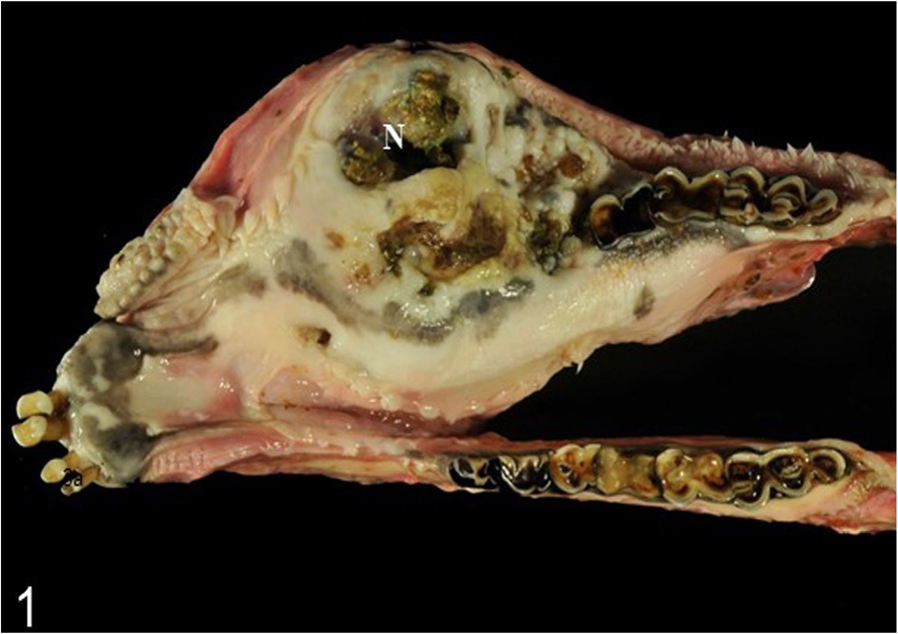
10 × 10 × 15 cm extensive and ulcerated mass in the right mandible. Ulcer (N), multifocal loss of teeth, and necrosis with plant material and cellular debris (N)

**Fig. 2:**
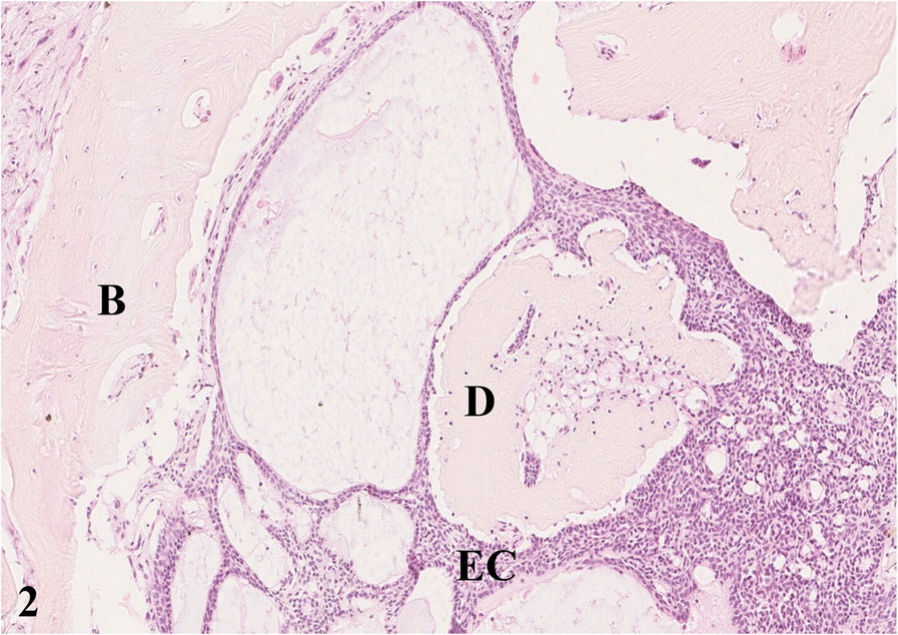
Mandibular bone (B) with infiltration by cords and islands of epithelial cells (EC) of odontogenic origin, surrounding islands of dentin (D) and amorphous pale eosinophilic matrix. HE staining, 10 x

**Fig. 3:**
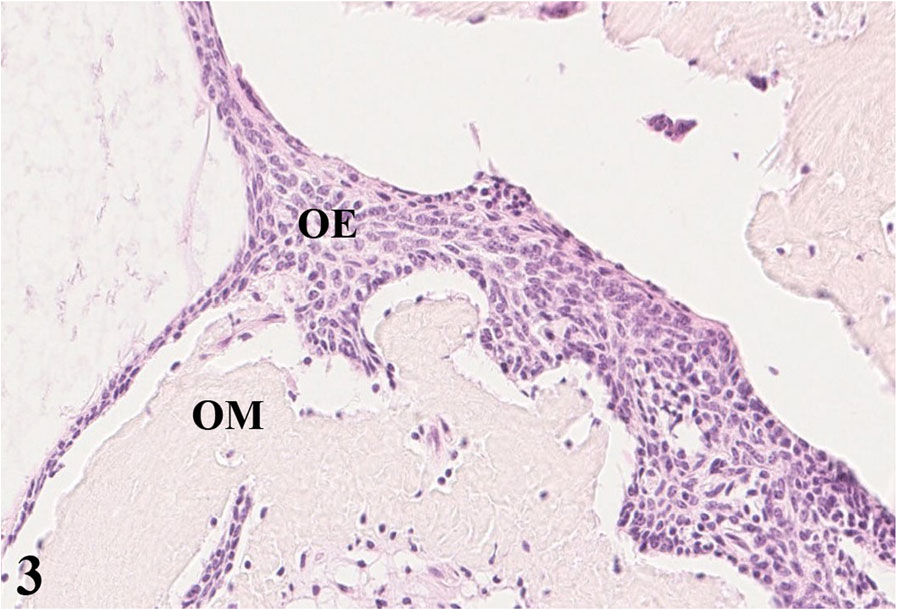
Cords and islands of epithelial cells of odontogenic origin (OE), surrounded by a matrix of odontogenic ectomesenchyme (OM), HE staining, 20x

**Fig. 4:**
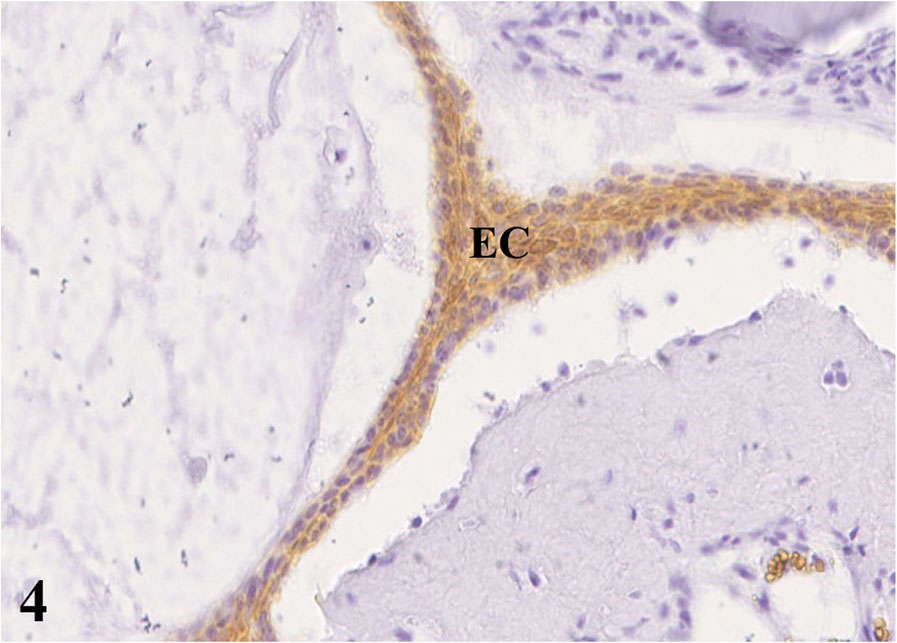
Immunohistochemistry for pan-cytokeratin, epithelial cells (EC) stain brown, 20x

 For pathological examination the head of an adult free-ranging female red deer (*Cervus elaphus elaphus*) was submitted. The animal and its fawn had been observed in June 2016 by rangers with a golf ball-sized mandibular mass. During the following months the animal had not been spotted again either by hunters or by camera trap. In June 2017, the animal was detected on trail camera records, tracked down, and shot due to its poor body condition. For pathohistological examination representative samples of the right mandible were fixed in 10 % neutral buffered formalin and demineralized in 10 % ethylenediaminetetraacetic acid (EDTA) for 10 days. Subsequently, the material was embedded in paraffin, sectioned at 4 µm and stained with hematoxylin and eosin (HE) as well as with trichrome mason stain, modified Gallego stain [5] and picrosirius red stain for demonstration of intercellular matrix. Immunohistological examination was done for expression of vimentin (mouse anti vimentin ABC monoclonal, DAKO® Hamburg, Germany) and pan-cytokeratin (mouse anti cytokeratin pan ABC monoclonal, OriGene Europe® Herford, Germany) with a biotinylated horse-anti-mouse-antibody (Vector Laboratories®) as secondary antibody to affirm either a mesenchymal or an epithelial origin of the mass. At gross examination, there was a 10 × 10 × 15 cm firm mass affecting the bone and connective tissue at the right mandible (Fig. 1). The gingival surface was ulcerated with multiple up to 2 × 2 × 1.5 cm wide foci of tissue necrosis containing plant fibers. Several teeth were missing (I2, I3, P2, P3, P4) in the right mandible (Fig. 1). At the ventral side of the mandible were two additional necrotic lesions of 2 × 1.5 × 1.5 cm with corresponding skin ulcerations. At the cut surface there were multiple different regions of firm (odontogenic) matrix mixed with inflammatory and necrotic areas. Histologically, an irregular proliferation of islands and cords of odontogenic epithelial cells (Figs. 2 and 3) (positive for pan-cytokeratin (Fig. 4), negative for vimentin) embedded in small amounts of a loosely arranged mesenchyme, and surrounding an unorganized osteoid-like material and a matrix of dentin (stained blue in trichrome masson stain, bright red in picrosiriusred stain, and yellow-greenish in Modified Gallego stain) between the bone trabecules was found. Epithelial cells were mostly cuboidal shaped with antibasilar nuclei. The cytoplasm of these neoplastic cells was bright eosinophilic. Anisocytosis and anisokaryosis were mild and the mitotic count was less than 1 per 10 high power fields. There were pinpoint foci of osteolysis surrounded by numerous osteoclasts. In areas of gingival ulceration plant fibers and bacterial colonies were detected on the surface, and in deeper areas coagulative necrosis with cellular debris admixed with infiltrates of neutrophils and plasma cells was present. The necrosis was surrounded by granulation tissue composed of fibroblasts (positive for vimentin), small vessels (positive for vimentin) and collagenous stroma. Due to these findings, the mass was diagnosed as odontogenic tumor with odontogenic ectomesenchyme, dental hard tissue formation, and secondary chronic purulent osteomyelitis. Considering the histomorphology of the tumor and that odontoameloblastoma as well as ameloblastic fibro-odontoma can still develop after completion of odontogenesis whereas complex odontomas are discussed to be hamartomas, odontoameloblastoma represents the most likely diagnosis for this case in the context of the animal’s age.

## Discussion and conclusions

 Odontogenic tumors have rarely been described in animal species with most frequent occurrence in dogs and cats. They must be differentiated from other oral neoplasms like melanoma or squamous cell carcinoma [6, 7]. Odontoameloblastoma are described rarely in different species, like guinea pigs, rats, and horse among others [3, 8]. For wildlife species such as deers, only one report on a compound odontoma in a 1.5-year-old white-tailed deer (*Odocoileus virginianus*) exists [9]. Regarding free-ranging or wildlife species, an odontoma in an African elephant (*Loxodonta africana*) [10] as well as some reports of odontomas in fish have already been described. The reports in fish comprise two cases of odontoma in clownfishs (*Amphiprion ocellaris*) and one case in a walleye (*Sander vitreus*) [11, 12]. Ameloblastic fibro-odontoma is reported in horses, dogs and cattle [13–15]. It is rarely diagnosed in wildlife species like llama (*Lama glama*) or in a cynomolgus macaque (*Macaca fascicularis*) [16, 17]. Other neoplastic diseases that must be considered as differentials are other odontogenic tumors like ameloblastoma and ameloblastic fibroma as well as tumors of mesenchymal origin like osteosarcoma or chondrosarcoma. In ameloblastic fibroma a minor involvement of epithelial cells is expected whereas ameloblastoma could be excluded considering the presence of odontogenic ectomesenchyme. Other neoplasms of mesenchymal origin could be excluded by their typical histomorphology (i.e. ostal or chondral tissue components in osteosarcoma/chondrosarcoma). Inflammatory processes, like mandibular osteomyelitis (lumpy jaw/actinomycosis) caused by *Actinomyces bovis* must be kept in mind as a differential diagnosis for neoplastic proliferations in ruminants. Overall, reports on inflammatory mandibular masses in fallow deer and roe deer (*Capreolus capreolus*) are more common than in red deer maybe because the former two species are farmed. A retrospective study from 2017 examined more than 1500 roe deer in Switzerland and detected actinomycosis and associated lesions in 1 % of the animals [18]. To our knowledge this is the first report of an ondontogenic tumor in red deer (*Cervus elaphus elaphus*). Odontoameloblastoma, like all odontogenic tumors, represent an uncommon neoplasia in most species but must be considered as a differential diagnosis in addition to the more common inflammatory or neoplastic diseases of the mandible even in wildlife species.

## Data Availability

The datasets used and/or analysed during the current study are available from the corresponding author on reasonable request.

## References

[1] Munday JS, Löhr CV, Kiupel M. Tumors of the alimentary tract. In: D Meuten J, editor. Tumors in Domestic Animals. 5th ed: Wiley Blackwell; 2016.

[2] Head WK, Cullen J, Dubielzig R, Else WR, Misdorp W, Patnaik AK, et al. Histological classification of tumors of the alimentary system of domestic animals2003.

[3] Wong HE, Hedley J, Stapleton N, Murphy B, Priestnall SL. Odontoameloblastoma with extensive chondroid matrix deposition in a guinea pig. Journal of veterinary diagnostic investigation: official publication of the American Association of Veterinary Laboratory Diagnosticians, Inc 2018;30(5):793–7. PubMed PMID: 30132419. Pubmed Central PMCID: PMC6505797. Epub 2018/08/23. eng. 10.1177/1040638718794784 PMC6505797 30132419

[4] Singh AK, Kar IB, Mishra N, Sharma P. Ameloblastic fibroodontoma or complex odontoma: Two faces of the same coin. National journal of maxillofacial surgery. 2016;7(1):92–5. PubMed PMID: 28163488. Pubmed Central PMCID: 5242084. 10.4103/0975-5950.196129 PMC5242084 28163488

[5] Afroze SN, Ramulu S, Rao GV, Taneeru S, Bashamalla R, Vadla P. Demystifying the nature of hard tissues in odontogenic tumors using Modified Gallego’s stain: A preliminary study. Journal of oral and maxillofacial pathology: JOMFP. 2018;22(3):448. PubMed PMID: 30651705. Pubmed Central PMCID: 6306605. 10.4103/jomfp.JOMFP_33_18 PMC6306605 30651705

[6] Dillehay DL, Schoeb TR. Complex odontoma in a horse. Veterinary pathology. 1986;23(3):341–2. PubMed PMID: 3727323. 10.1177/030098588602300322 3727323

[7] Walsh KM, Denholm LJ, Cooper BJ. Epithelial odontogenic tumours in domestic animals. Journal of comparative pathology. 1987;97(5):503–21. PubMed PMID: 3316314. 10.1016/0021-9975(87)90002-8 3316314

[8] Murphy B, Bell C, Koehne A, Dubielzig RR. Mandibular odontoameloblastoma in a rat and a horse. Journal of veterinary diagnostic investigation: official publication of the American Association of Veterinary Laboratory Diagnosticians, Inc. 2017;29(4):536–40. PubMed PMID: 28545325. 10.1177/1040638717711996 28545325

[9] Fitzgerald SD. Clinical challenge. Diagnosis: oral mass; compound odontoma. Journal of zoo and wildlife medicine: official publication of the American Association of Zoo Veterinarians. 2000;31(3):419–21. PubMed PMID: 11237154. 10.1638/1042-7260(2000)031[0419:CC]2.0.CO;2 11237154

[10] Raubenheimer EJ, van Heerden WF, Turner ML, Mare LK (1989). Odontoma in an african elephant (Loxodonta africana). J S Afr Vet Assoc.

[11] Coffee LL, Bogdanovic LB, Cushing TL, Bowser PR. Pharyngeal odontoma in an adult walleye (Sander vitreus). Veterinary pathology. 2013;50(3):483–7. PubMed PMID: 22610032. 10.1177/0300985812446149 22610032

[12] Vorbach BS, Wolf JC, Yanong RP. Odontomas in two long-finned ocellaris clownfish (Amphiprion ocellaris). Journal of veterinary diagnostic investigation: official publication of the American Association of Veterinary Laboratory Diagnosticians, Inc. 2017:1040638717729726. PubMed PMID: 28906183. 10.1177/1040638717729726 PMC6504150 28906183

[13] Huang P, Bell C, Wallace V, Murphy BG. Mixed odontogenic tumors in four young dogs: ameloblastic fibroma and ameloblastic fibro-odontoma. Journal of veterinary diagnostic investigation: official publication of the American Association of Veterinary Laboratory Diagnosticians, Inc. 2019;31(1):98–102. PubMed PMID: 30451090. Pubmed Central PMCID: PMC6505756. Epub 2018/11/20. eng. 10.1177/1040638718812936 PMC6505756 30451090

[14] Knowles S, Blas-Machado U, Butler AM, Gomez-Ibañez SE, Lowder MQ, Fayrer-Hosken RA. Ameloblastic fibro-odontoma associated with a retained molar in an Oldenburg mare. Journal of veterinary diagnostic investigation: official publication of the American Association of Veterinary Laboratory Diagnosticians, Inc. 2010;22(6):987–90. PubMed PMID: 21088190. Epub 2010/11/23. eng. 10.1177/104063871002200625 21088190

[15] Tetens J, Ross MW, Sweeney RW. Rostral mandibulectomy for treatment of an ameloblastic fibro-odontoma in a cow. Journal of the American Veterinary Medical Association. 1995;207(12):1616-7. PubMed PMID: 7493903. Epub 1995/12/15. eng. 7493903

[16] Davis JA, Banks ER, Young D (1988). Ameloblastic odontoma in a cynomolgus monkey (Macaca fascicularis). Lab Anim.

[17] Step DL, Ritchey JW, Drost WT, Bahr RJ. Ameloblastic odontoma in the mandible of a llama. The Canadian veterinary journal = La revue veterinaire canadienne. 2003;44(10):824–7. PubMed PMID: 14601679. Pubmed Central PMCID: PMC340299. Epub 2003/11/07. eng. PMC340299 14601679

[18] Pewsner M, Origgi FC, Frey J, Ryser-Degiorgis MP (2017). Assessing fifty years of general health surveillance of roe deer in Switzerland: A retrospective analysis of necropsy reports. PloS one.

